# Notes and News

**Published:** 1891-07-11

**Authors:** 


### Notes and News.

Collected and Communicated.

The foundation-stone of the new children's ward for Lincoln Hospital was quietly laid last month by Mr. Joseph Ruston, the donor of the building. At the west of the existing hospital a new wing will be erected, containing an out-patients' department with almost precisely similar accommodation to that which is going to be destroyed, and as shown by the admirable sketch exhibited on Saturday by the architect, Mr. W. Watkins, the building will have a flat roof, surmounted by a balustrade, which will form a pleasant promenade and airing ground for convalescent patients in summer time.

The Duchess of Teck, the Earl of Stafford, and others honoured the annual meeting of the East-end Mothers' Home by their attendance. The number of patients for the year ending April was 169, of whom 116 were in-patients and 27 were attended at their own homes. It was the wish of the benevolent ladies who had done so much in behalf of the Home, to start an out-patient department, and Mrs. Joicey had given £100 with that object, and had guaranteed £50 a-year in future. With regard to the financial position of the Home, the income of the year had not been sufficient to render it possible to pay off any of the bank loan of £1,500 for the purchase of the freehold premises.

Until this year the people of Dudley took but small interest in the Hospital Saturday movement. The result has been the collection of £9 or £10 from probably between 10,000 or 15,000 people who perambulate the streets. It is altogether unworthy of Black Country people, seeing that the Guest Hospital opens its doors to the great industrial centre which has Dudley as its metropolis. This year the collection was taken up in a more hearty style, and nearly three times the amount of money raised than has been the case in previous years. This should be a valuable hint to the authorities to make Hospital Saturday a more important day than it is at present. The nurses went in for it with a zest that did them the greatest possible credit.

The new west wing which has just been added to the Hospital for Incurables at Morningfield, Aberdeen, was opened on June 29th, when the Committee of Managers appointed in connection with the conduct of the institution paid a formal visit for the purpose of inspecting the addition. The architects are Messrs. W. Henderson and Sons. In the new wing, which is two storeys high, there is accommodation for 26 additional patients. The new building stands apart from the original portion of the hospital, with which it is connected, however, by a corridor six feet wide. There are four wards, nurses' rooms, bath-rooms, &c., in the new block, which is provided with broad, airy staircases, and passages. The wards, which are splendidly lighted, are 14 feet high from floor to ceiling.

A new cure for tuberculosis is reported from Paris. It is our familiar friend chloride of zinc impressed into a new service. There will be time enough for criticism when a sufficient number of verified facts has been accumulated.

Some excellent articles have been appearing in one of the best American papers, the *Nation*, on the Parisian hospitals and charitable institutions. They are by a lady, Miss McIlvaine, though for a long time the editor was under the belief that they were written by a man, so masterly was the grasp of the whole subject that the author displayed.

The Duke of Westminster, accompanied by the Duchess, will visit the National Hospital for the Paralysed and Epileptic (Albany Memorial) Queen Square, Bloomsbury, on Saturday the 18th inst. to inaugurate the new surgical wing. It was hoped the building would have been fully completed by the day named, but the carpenters and joiners' strike has prevented this, and the opening ceremony therefore will be formal.

For the successful treatment of his wife in a dangerous surgical operation performed by Dr. Michelsen, of Wiesbaden, Herr von Donner, a merchant of Hamburg, has placed at the disposal of the authorities in Hamburg, as a thank-offering, the sum of two million marks. This money is to be expended in the erection of a hospital in Hamburg, in which Dr. Michelsen is to be installed as chief physician. The building will take two years to complete, and at the expiration of that period Dr. Michelsen will leave the hospital at Wiesbaden for the medical establishment in Hamburg.

The medical officer of a certain lunatic asylum proposes to improve on Professor Garner's experiments in monkey speech. Professor Garner caught in a phonagraph the cries of monkeys when thirsty, hungry, and when afraid. He then went home and turned on the phonograph and learnt the three sounds; he then visited a different monkey house and conversed with the inmates in their own language, and they obviously understood him. Our asylum friend says that idiot children have just these three cries also, so the Darwinian theory receives confirmation, since the language of the lowest human and the highest ape is similar.

On Saturday, 4th inst., the Lady Mayoress visited the Royal Albert Orphan Asylum, near Bagshot, for the purpose of presenting the prizes. This asylum was established in 1864 for children of both sexes, and the elections are conducted on non-canvassing principles, by which expense is saved to the friends of the orphans. There is room for 220 boys and girls, but at present, owing to want of funds, there are about 50 short of this number. The children are all taught trades under competent tradesmasters, and an exhibition of their work was of considerable interest, consisting of clothes of various kinds, carpenter's work, boots, &c., together with produce from the boys' farms. It should be mentioned that the whole of the clothes of the establishment are made by the children themselves under the direction of teachers. The general education is not, however, sacrificed to their industrial training, as is proved by the fact that at the last visit of the inspector 90 per cent. of the boys passed their various standards. After the presentation of prizes there was a musical drill of the girls, followed by a very amusing model debate by some of the boys upon "Getting on in the World." General Stotherd, C.B., the Chairman of the local committee, then thanked the Lady Mayoress for her kindness in presenting the prizes and shortly sketched the objects of the Asylum which he said was very much in want of funds. Among the visitors were Lady W. Seymour, Hon. Mrs. Eliot, Sir W. Marsh, K.C.M.G., Major-General Stotherd, C.B., Colonel Wavel, Dr. Collingwood, and F. H. Goldney, Esq. The boys' band played a selection of music during the afternoon.

